# Tuning the stator subunit of the flagellar motor with coiled‐coil engineering

**DOI:** 10.1002/pro.4811

**Published:** 2023-12-01

**Authors:** Pietro Ridone, Daniel L. Winter, Matthew A. B. Baker

**Affiliations:** ^1^ School of Biotechnology and Biomolecular Science UNSW Sydney Sydney Australia

**Keywords:** bacterial motility, coiled‐coil, flagellar motor, molecular motor, transmembrane

## Abstract

Many bacteria swim driven by an extracellular filament rotated by the bacterial flagellar motor. This motor is powered by the stator complex, MotA_5_MotB_2_, an heptameric complex which forms an ion channel which couples energy from the ion motive force to torque generation. Recent structural work revealed that stator complex consists of a ring of five MotA subunits which rotate around a central dimer of MotB subunits. Transmembrane (TM) domains TM3 and TM4 from MotA combine with the single TM domain from MotB to form two separate ion channels within this complex. Much is known about the ion binding site and ion specificity; however, to date, no modeling has been undertaken to explore the MotB‐MotB dimer stability and the role of MotB conformational dynamics during rotation. Here, we modeled the central MotB dimer using coiled‐coil engineering and modeling principles and calculated free energies to identify stable states in the operating cycle of the stator. We found three stable coiled‐coil states with dimer interface angles of 28°, 56°, and 64°. We tested the effect of strategic mutagenesis on the comparative energy of the states and correlated motility with a specific hierarchy of stability between the three states. In general, our results indicate agreement with existing models describing a 36° rotation step of the MotA pentameric ring during the power stroke and provide an energetic basis for the coordinated rotation of the central MotB dimer based on coiled‐coil modeling.

## 1 INTRODUCTION

The bacterial flagellar motor (BFM) is a complex nanoscale engine that converts ion flux into the rotation of a flagellum to propel bacteria through their surroundings (Mondino et al., 2022; Nakamura & Minamino, 2019). A crucial component of this complex is the stator subunit which is responsible for the torque‐generating step of the motor (Mandadapu et al., 2015; Nirody et al., 2017). In *Escherichia coli*, the stator subunit is in fact composed of two transmembrane (TM) proteins (MotA and MotB) which assemble around the basal rotor of the flagellar motor and form a proton channel at their interface (Guo & Liu, 2022). The inward flux of ions across the complex is efficiently coupled to a mechanical stimulus applied onto the rotor protein FliG which ultimately drives the rotation of a single flagellum (Hu, Santiveri, et al., 2022). Understanding the fundamental biophysical principles behind biological rotary motors is of interest in synthetic biology and medicine where this efficient mechano‐electric coupling can be employed in biosensing and nanotechnology applications or targeted in antibacterial strategies.

The structure of the full MotA_5_MotB_2_ complex was solved in 2020 (Deme et al., 2020; Santiveri et al., 2020), revealing that the stator complex likely acted as a rotary motor itself. Soon after, cryoET work observed conformational rearrangements of the switch complex to enable bidirectional coupling of the rotor to a rotating stator (Chang et al., 2020). Recent structural work has identified the basis for ion selectivity and many catalytic residues involved in the process (Hu et al., 2023). The current consensus model is that a TM pentameric ring of MotA subunits rotates around a central dimer stalk of MotB TM domains (Hu, Popp, et al., 2022; Rieu et al., 2022). The model details the conformational waypoints through which the MotA subunit passes during the gating cycle of the stator, but our understanding of the dynamics, in general, is limited, and in particularly for the MotB subunit central stalk. The central MotB dimer is important because it harbors the catalytic center. It has been proposed to rotate in place between protonated and non‐protonated states (Rieu et al., 2022). Previous work suggested that the MotB TM domains exchange their interface contacts during gating and may pivot along their helical axis (Braun & Blair, 2001), in a similar fashion to the proposed mechanism for the homologous TolR protein (Zhang et al., 2011). The dimerization of MotB at the TM domain was shown to be essential for motility as modifications to TM residues that restrict or alter the conformational space of the dimer (e.g., cysteine crosslinking and tryptophan‐scanning mutagenesis) generally diminished motility (Braun & Blair, 2001; Sharp et al., 1995; Zhang et al., 2011).

The MotB stalk appears to assemble as a dimeric parallel coiled‐coil in recently published structures (Deme et al., 2020; Santiveri et al., 2020). Coiled‐coils are well‐characterized structural motifs where alpha‐helices interact to form dimers or higher order oligomeric states (Lupas & Bassler, 2017). A repeating seven residues‐long “heptad” motif with alternating hydrophobic (*h*) and polar (*p*) residues forming the general pattern *hpphppp* along the alpha‐helices results in a core interface where the hydrophobic residues engage in knob‐into‐hole (KIH) interactions. The sequence‐structure relationship of coiled‐coils is well understood, and design principles have been established to create synthetic coiled‐coils. Dimers are a specific example: it has been shown that isoleucine and leucine in positions *a* and *d* of a repeating heptad *abcdefg* (corresponding to the pattern *hpphppp*) greatly favor dimerization over other oligomeric states, whereas isoleucine at both positions favors trimers (Fletcher et al., 2012). Moreover, the structure of coiled‐coils can be parametrically modeled, which greatly accelerates the calculation of their physical properties, such as interaction strengths (Crick, 1953). Parametric modeling entails the use of very few parameters (the radius, interface angle, and pitch of a coiled‐coil) to describe the entire backbone structure. Parametric modeling, in combination with the BUDE (Bristol University Docking Engine) (McIntosh‐Smith et al., 2015) forcefield, has been successfully applied to predict the structures of natural and synthetic coiled‐coils (Wood et al., 2014; Wood & Woolfson, 2018).

In this study, we investigated the dimerization interface of MotB using parametric modeling to represent the MotB dimer as a dynamic coiled‐coil. This simplification of the MotB structure enables the rapid calculation of the total energy of the system to discriminate between stable and unstable conformations. From these results, we rationally designed stator sequence variants where we varied isoleucine, leucine, and alanine in critical locations across the coiled‐coils to stabilize or destabilize the dimer. We then tested these in vivo to better understand how MotB dimer stability may relate to the conformational dynamics of the stator complex during its gating cycle. By testing the in vivo motility of MotB variants predicted to differ in stable and unstable conformational states, we aimed to identify which coiled‐coil conformations were correlated with motility, and to inform the structural changes undertaken by *Ec*MotB and its extant homologs during their gating cycle.

## 2 RESULTS

**FIGURE 1:**
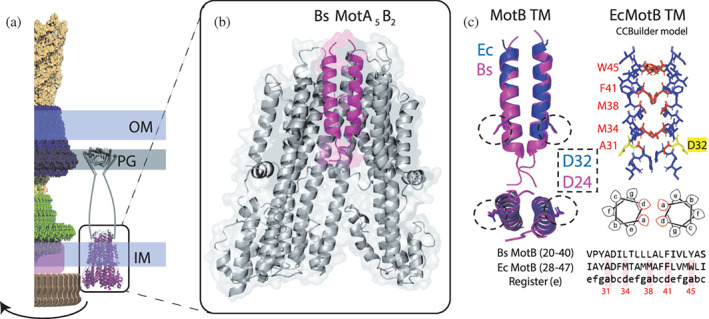
Modeling the transmembrane (TM) domain of MotB as a dimeric coiled‐coil. (a) The stator subunit of the bacterial flagellar motor (BFM) of *Escherichia coli* (MotAB) drives the rotation of the large rotor complex by converting the proton motive force into mechanical torque. The BFM is located across the inner and outer membranes (IM, OM) and peptidoglycan layer (PG) and powers the rotation of a flagellum that propels the cell. The stator unit MotA_5_MotB_2_ acts as an ion channel and each MotA has four TM domains while MotB has a single TM domain and exists as a dimer in the centre of the complex. (b) The cryo‐EM structure of *B*. *subtilis* MotA_5_B_2_[PDB 6YSL] (left) was used as the starting point for *E*. *coli* MotB TM modeling. The MotB dimer is shown in pink. (c) A CCBuilder model of the homologous sequence from *E*. *coli* was generated (blue) and aligned to the reference *Bs*MotB dimer (purple). A coiled‐coil model in the *e* register was selected based on the orientation of the catalytic aspartates D32 (*E*. *coli*) and D24 (*B*. *subtilis*) (middle, dotted circles in the side, and bottom views of the TM domain). Residues A31, M34, M38, F41, and W45 were found to be at the coiled‐coil interface (right), corresponding to positions *a* and *d* of the heptad repeat able to participate in knob‐in‐kole interactions (labeled in red on the atomic model and heptad diagram underneath). An alignment of the two homologous amino acid sequences and the selected register is shown on the bottom‐right, with *E*. *coli* residues at the *a* and *d* positions highlighted in red and numbered.

We modeled the *Ec*MotB TM domain as a parallel coiled‐coil based on the most recent structures of MotB from *Bacillus subtilis* (PDBID: 6YSL), *Clostridium sporogenes* (PDBID: 6YSF), and *Campylobacter jejuni* (PDBID: 6YKM) (Deme et al., 2020; Santiveri et al., 2020) (Figure 1). A set of ISAMBARD models of the 20‐residue long *Ec*MotB peptide IAYA**D**FMTAMMAFFLVMWLI (corresponding to residues 28–47 of WT *Ec*MotB; the catalytic residue D32 is indicated in bold) with varying interface angles were aligned to the structures to formally identify the “register” of the MotB coiled‐coil, that is, what positions in the coiled‐coil heptad each residue occupies. Based on RMSD calculations and the orientation of the catalytic aspartate (D32), we established that the 20‐residue long MotB dimer stalk is best described as a coiled‐coil starting at position *e*. In other words, residues A31, M34, M38, F41, and W45 occupy positions *a* and *d* along the coiled‐coil, forming the core interface. These results agree with previous reports where mutation of these residues to cysteine led to crosslinking between the two MotB subunits, indicating proximity (Braun & Blair, 2001). Further analysis using CCBuilder of the peptide IAYADFMTAMMAFFLVMWLI as a parallel dimeric coiled‐coil starting at register *e* indicated that the five core residues could form KIH interactions.

**FIGURE 2:**
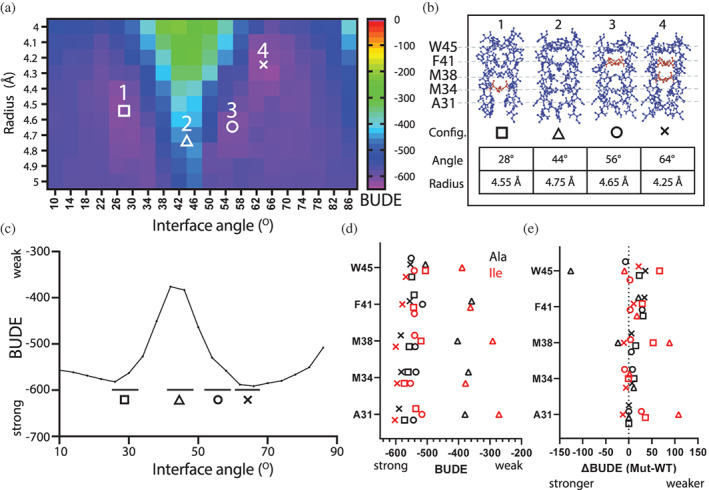
Characterization of the MotB transmembrane coiled‐coil interaction energy. (a) Free energy (BUDE) between MotB dimer coils represented as heatmap indicating BUDE value versus interface angle and radius. Four configurations are highlighted by symbols. (b) Atomic models of the MotB coiled‐coil for four configurations (28°, 44°, 56°, and 64°, identified by the square, triangle, circle, and cross symbols respectively). The main amino acid residue responsible for KIH interactions are labeled in red. (c) 2D BUDE profile calculated from mean across 10 radii values from (a). (d) predicted BUDE energy changes at each of the four configurations (symbols) after amino acid replacement at positions *a* and *d* of the MotB coil with amino acids alanine, (e) ∆BUDE (BUDE^mut^ − BUDE^wt^) for each of four configurations. The BUDE value calculated at interface angles 28°, 44°, 56°, and 64° is calculated as the average of the two neighboring data points above the black horizontal bars. Substitutions are indicated by color (Ala, black) or isoleucine (Ile, red).

In light of these results, we decided to model MotB as a dimeric coiled to inform our in vivo mutagenesis experiments. To this end, rather than simply considering the most stable conformation calculated by ISAMBARD, we considered the conformational space of MotB by generating coiled‐coil structural models with varying interface angles and radii and then, for each combination of the two parameter values, calculating the total energy of the system using the BUDE forcefield (hereafter, BUDE) (Figure S1A). This in silico conformational screen revealed the potential existence of wells (more stable configurations) and peaks (unstable configurations) across the energy landscape of the WT *Ec*MotB (Figure 2a). Interestingly, the deepest stability well (#4, Figure 2b) did not correspond to the configuration adopted in the cryo‐EM structures (#1, Figure 2b).

Next, we considered strategic point mutations of coiled‐coils to investigate if the stability of the MotB dimer would change after replacing the key *a* and *d* residues forming the core interface. We considered the design rules of synthetic coiled‐coils and hypothesized that alanine mutations would systematically weaken the coiled‐coil interaction by disrupting the hydrophobic KIH interactions. In contrast, isoleucine and leucine mutations would lead to more stable KIH interactions and thus a more stable coiled‐coil (when isoleucine and leucine occupy positions *a* and *d*) or to steric clashes (when isoleucine occupies both positions *a* and *d*). Therefore, core residues were systematically mutated in silico to alanine, isoleucine, or leucine at positions *a* and *d* in over 200 permutations (Section 4 and Table S1). In addition, we also considered the M38G mutation that appeared to disrupt KIH interactions in CCBuilder analyses. In total, a library of 221 sequence variants was generated in silico.

To test the above hypothesis in silico, we repeated the conformational analysis using ISAMBARD for each MotB variant modeled as a coiled‐coil and compared the energy landscape of each sequence variant to the WT sequence. Different mutations resulted in a shift of the energy wells toward different interface angles and radii values and, in some extreme cases, to the absence of stability wells. The energy peaks corresponding to potential inaccessible conformations also differed. Altogether, the results indicate that the selected mutations in the core of the MotB dimerization interface could affect its accessible conformational space and, presumably, affect cell motility. Further to this analysis, the three‐dimensional (3D) BUDE energy landscapes were collapsed into two‐dimensional (2D) energy profiles by averaging the energy values for radii between 4 and 5 Å (Figure 2c). This range of radii values in BUDE encompassed the radii observed in the reference structures (4.7–4.9 Å) and contained most of the energy wells and peaks of interest to compare sequence variants. In these dimensionally reduced datasets, we subsequently considered the interface angle parameter as the main variable affecting the energy of the coiled‐coil system.

We next sought to select a subset of mutants to verify whether the differences in calculated energy profiles for MotB sequence variants correlated with BFM activity. Comparison of the 2D BUDE energy profiles suggested that single mutations could be sufficient to alter the accessible conformational space of MotB, and therefore affect cell motility (Figure 2c,d). We selected 9 single mutants (Figure S1B,C) and an additional 10 double mutants and 4 triple mutants (Figure S2) that showed varied energy profiles for in vivo experimentation. We restricted our in vivo testing to variants displaying alanine and/or isoleucine replacements.

We recombinantly expressed the selected sequence variants of MotB in an *E*. *coli* Δ*motAB* background strain and assessed the motility of bacteria on swim plates (Figure S2A,B).

A comparison of the mean 2D BUDE profiles of all motile and non‐motile variants suggested that the stability of the coiled‐coil around the interface angle of 28°, where M34 plays a key role in KIH interaction, was crucial for function (Figure S2C). The influence on the total energy from mutation at that site, adjacent to the catalytic aspartate residue (D32), appears to be correlated with a motile phenotype. When comparing the 2D BUDE profiles of these MotB variant tested in vivo, we noticed a similarity in the shapes of the BUDE profiles of the motile variants, whereas the profiles of the non‐motile variants diverged substantially (Figure S2D). Aligning the profiles with respect to their peak values (BUDE^max^) revealed additional differences between motile and non‐motile profiles than the strength of interaction at the 28° configuration alone. The BUDE gap between the minimum value of each profile and the peak corresponding to the 44° configuration appeared to be similar across the motile variants.

These results suggest that a combination of energetic parameters contained within the 2D profiles could be descriptive of functional *Ec*MotB variants and that the relative stability of each configuration needs to be maintained to operate the *E*. *coli* stator.

**FIGURE 3:**
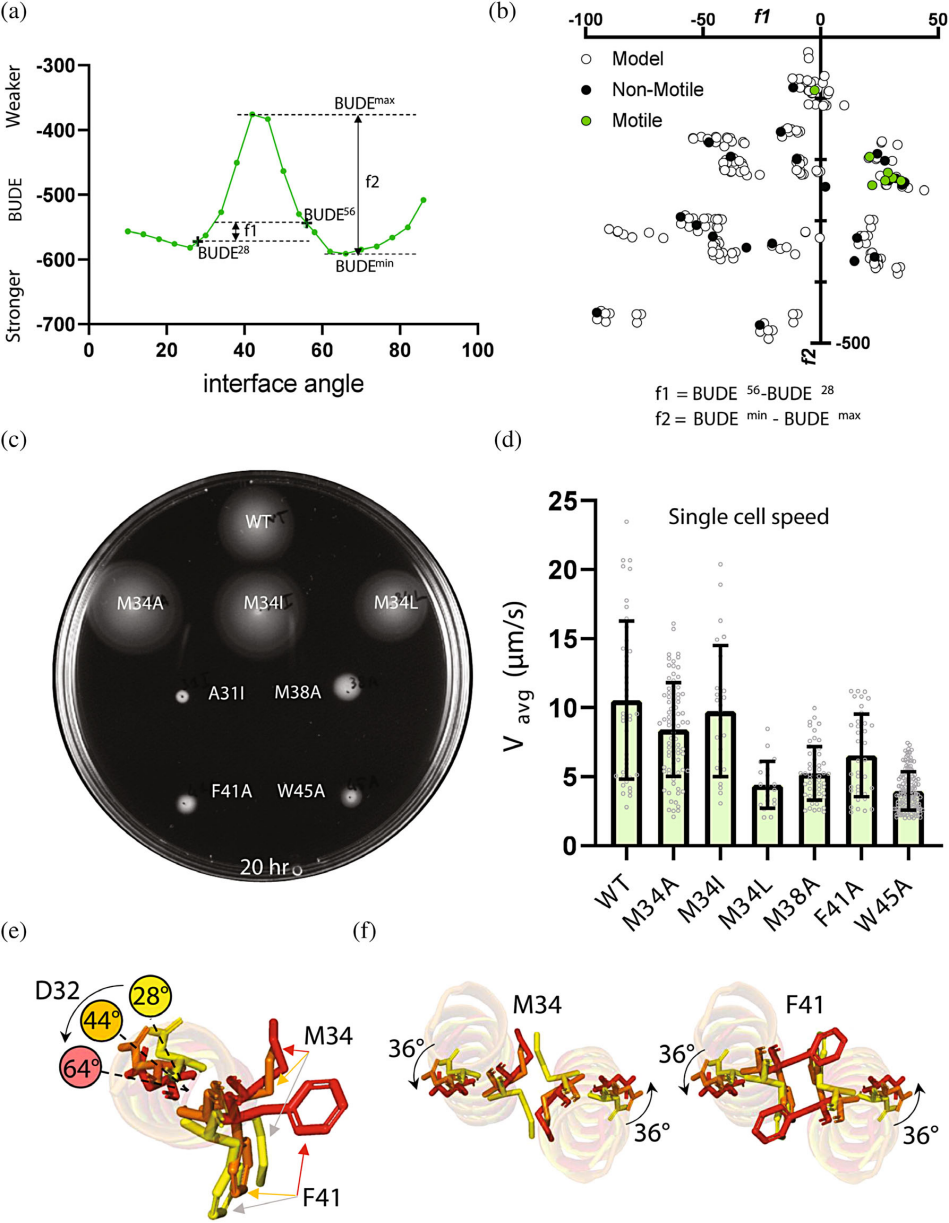
Testing coiled‐coil variants in *Escherichia coli* and parametrization of 2D BUDE profiles. (a) The *f*1 and *f*2 parameters visualized on the 2D BUDE profile of *E. coli* WT MotB. (b) Individual variants plotted on a graph (*X* = *f*1 and *Y* = *f*2). Among the 221 variant, 28 were tested and 7 were found to be motile (green). The formulas defining *f*1 and *f*2 are reported below the graph. (c) Swim plate assay of *E. coli* Δ*motAB* + pDB108 expressing WT MotA and either WT (top) or mutant MotB's on LB agar 0.3% supplied with 0.1% arabinose and chloramphenicol after 24 h incubation at 30°C. WT MotB, M34A, M34I, M34L, M38A, F41A, and W45A were found to be motile. The non‐motile MotB variant A31I is shown here for comparison. (d) Single *E. coli* cell swimming speed measured in motility buffer (*n* = 42; 83; 25; 22; 57; 37; and 157). Bars indicate Mean speed (μm/s) + SD. (e) Top view of a single MotB helix in a coiled‐coil at interface angles 28°, 44°, and 64°, each colored in yellow, orange, and red respectively. A curved arrow indicates the direction of rotation. Residues D32, M34, and F41 are shown for each angle. (f) Top views of the MotB dimeric coiled‐coil at the three interface angles described in E. Residues M34 (left) and F41 (right) are shown along with D32.

We captured the features associated with motile variants by defining two metrics, *f*1 and *f*2 (Figure 3a), where *f*1 = BUDE^56°^ − BUDE^28°^ and *f*2 = BUDE^min^ − BUDE^max^. The first metric, *f*1, is the BUDE energy difference between the BUDE energy at an interface angle of 56° and the BUDE energy at an interface angle of 28° (Figure S2C). This metric accounts for the BUDE energy difference at 28° associated with motile and non‐motile variants at 28° and also the BUDE energy at 56° which is predicted to form a KIH interaction with the conserved residue F41 (Figure 2b, “circle” configuration). Residue F41 appeared to have the more consistent value across the profiles of all variants (Figure S3). With the *f*2 metric we measured the range between the minimum and maximum BUDE values across the profile, or the energy barrier separating the energy wells. Separating all the variants modeled in silico based on these two metrics revealed a cluster of optimal *f*1 and *f*2 values for the motile variants of *E*. *coli* MotB (Figure 3b).

A total of 30 different mutants, also including leucine replacements of KIH residues which clustered with the motile variants were prepared and tested in vivo (Table S1) to identify motile variants and determine which configurations were correlated with motility. A total of six MotB variants were found to be motile: M34A, M34I, and M34L which produced swim rings similar in size to WT while M38A, F41A, and W45A displayed reduced motility (Figure 3c). The same trend in phenotypes was displayed at the single cell level in the free‐swimming assay (Figure 3d). These experiments suggest that mutations targeting the C‐terminal half of the coiled‐coil have a negative effect on motility while the M34 site on the other hand is tolerant to hydrophobic replacements. A detailed analysis of the side chains of residues M34 and F41 in three configurations suggests that these residues change orientation at different stages of the transition between 28° and 64° (Figure 3e,f). After the first residues turn (M34), F41 experiences steric hindrance against its counterpart F41 and destabilization of the coiled‐coil before reorienting.

Further comparison of the 2D profiles of all point mutants suggested a pattern of specific interface angles, or configurations, associated with motility (Figure S4). All the *Ec*MotB variants found to be motile had the total energies associated with the 66° (cross), 28° (square), 56° (circle), and 44° (triangle) configurations in ascending order of magnitude. The exceptions to this pattern were the single mutants A31I, F41I and M38G, which featured destabilized 44°, 28°, and 28° configurations, respectively, and the double mutants A31I M34I and A31I M38A which featured a destabilized 44° configuration. This suggested that the relative stability of the 28°, 56°, and 64° configurations play a role in determining the function of MotB and that this pattern may reflect the order of configurations undertaken by WT MotB during its gating.

When all variants tested in vivo were modeled in the 28°, 56°, and 64° configurations, it appeared that many of the non‐motile variants had missing key KIH interactions that were otherwise present in the WT MotB model (Figure S5). Among all variants, 28 models out of 29 (96.5%) could be predicted to be non‐motile based only on their KIH patterns at the three configurations. All motile variants displayed KIH patterns across configurations as those seen in WT.

Taken together, these data suggest that MotB uses KIH interactions during its gating cycle to stabilize specific coiled‐coil configuration and that the transition between these configurations is crucial for stator function. It is possible that the coiled‐coil domain of MotB rotates between these configurations in a specific order during the stator gating cycle.

**FIGURE 4:**
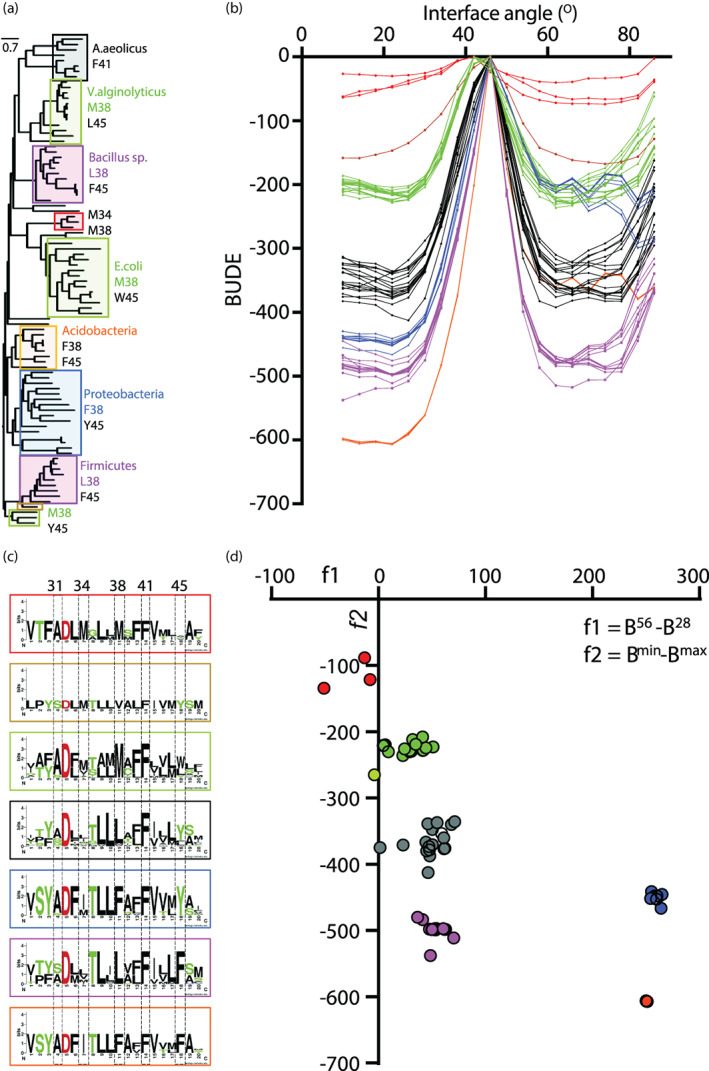
BUDE analysis of extant MotB sequences. (a) Phylogeny of extant, full‐length MotBs. Clades are labeled by colored boxes corresponding to the clusters highlighted in b. A representative member of each clade, or representative bacterial phylum, are shown next to each box. The conserved amino acids indicative of each clade are shown next to each box and numbered according to *E*. *coli* residues. A detailed phylogeny is provided in Figure S7. (b) 2D BUDE plots of extant WT MotB variants, translated by their BUDE^max^ values. (c) Consensus logos for each plot in B. Each logo is inscribed in a box matching the color of its respective plots in A. Knob‐in‐hole residues at positions homologous to *Ec*MotB 31, 34, 38, 41, and 45 are highlighted in vertical gray boxes. (d) Clustered populations of extant MotB stators plotted on a graph (*X* = *f*1 and *Y* = *f*2). The formulas used to calculate *f*1 and *f*2 for each homolog are provided below the *X* axis.

Finally, we explored a larger set of homologous MotB sequences to determine whether our observations were applicable to other extant motile bacteria. We generated a 91 member phylogeny of MotB using RAxML (Stamatakis, 2014) across diverse clades (Figures 4A, S6, and S7) and carried out the same BUDE analysis on 70 representative MotB sequences across the phylogeny (Figures S6A and S7) to produce a 2D BUDE energy profile for each of the 20 residues‐long TM domain modeled as a coiled‐coil (Figure S6B). To compare the energy profiles to each other and to the phylogenetic clustering, we translated each BUDE profile relative to its BUDE^max^ value (Figure 4b). This analysis of the energy profiles distinguished seven distinct groups (Figure 4c) that matched the phylogenetic clades (Figure 4a), and each displaying one or more conserved amino acid residues among the representative positions *a* and *d* of their heptad repeats, evidenced by their consensus logos (Figure 4c). These groups clustered around specific values of the *f*1 and *f*2 metrics (Figure 4d). Out of these 70 sequences, 66 were found to have *f*1 > 0, while only a subset had *f*2 > 0 (Figure S6C). Clusters were separated by the identity of their residue 38 (position *a*) and shared the conserved D32 and F41 (*d*) residues. Positions 31 (*a*), 34 (*d*) and 45 (*a*) also displayed unique residues in specific clusters (Figure 4a,c).

## 3 DISCUSSION

In this study we aimed to illustrate the conformational changes undergone by MotB during the gating cycle of the stator by constructing several MotB stator variants based on in silico designs, following coiled‐coil design principles, and then by assessing their capacity to drive swimming in vivo in *E*. *coli*. Because the residues targeted by mutagenesis were at the hydrophobic interface of the coiled‐coil structure, we assumed that the effects of amino acid replacement would be confined to MotB and its dimerization and thus we restricted our analyses to symmetric configurations of the MotB dimer. In other words, we assumed that movement (i.e., rotation) of one MotB subunit was always accompanied by the same movement of the second subunit. We sought to examine how motility of the overall stator complex was disrupted when assembly of the coiled‐coil via KIH was altered. After finding several energetic minima from ISAMBARD analyses (Figure 2), we focused on variations of the interface angle between the two MotB alpha‐helices, which correspond to the rotation of the alpha‐helices relative to each other and the possible transitions between these stable conformations. This type of rotation is known as a “register shift” because the interface angle is related to the coiled‐coil register and register shifts have been shown elsewhere to underpin biological processes such as dynein recruitment (Choi et al., 2011; Noell et al., 2019) and the activity of proteasomal ATPase (Snoberger et al., 2018).

**FIGURE 5:**
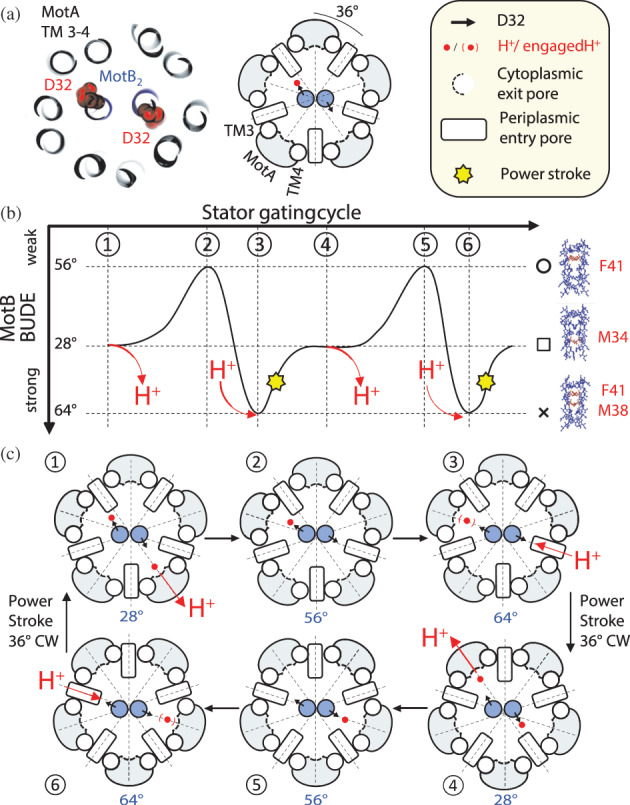
Rotary model showing the role of MotB coiled‐coil conformations in the mechanochemical cycle of the stator complex. (a) Section of the *Bs*MotAB [PDB 6YSL] structure highlighting the central MotB dimer (blue) and transmembrane (TM) helices TM3 and TM4 of MotA (gray) interfacing the MotB dimer. A sphere model (red) indicates the position and orientation of the catalytic aspartate in MotB. A cartoon representation of the structure and legend are shown on the right. The dashed lines below separate the stator cross‐section in 10 identical 36° sectors. (b) MotB TM BUDE fluctuations between three configurations during the gating cycle of the stator. The red arrows indicate binding or unbinding of a proton at the left or right aspartate (H‐ or ‐H, respectively). A yellow star indicates the power stroke and 36° clockwise rotation of the stator. Configuration symbols and representative knob‐into‐hole interactions are also shown on the right of the model. (c) Six stages of the mechanochemical cycle model described in B. A proton is transferred into the cytoplasm through an exit pore immediately after a power stroke (1). A change in the MotB TM configuration follows, which rotates 36° counterclockwise from the 28° (1) to the 64° configuration (3), via the 56° configuration (2). Once in the 64° configuration, the aspartate on the left subunit engages MotA at the TM3‐4 interface in proximity of the cytoplasmic exit pore (3). A proton then enters from the opposite periplasmic entry pore to bind the unprotonated aspartate causing a conformational change of the coiled‐coil and the coordinated rotation of the MotB dimer and MotA pentamer by 36° clockwise (a power stroke). This rotation restores the coiled‐coil to the 28° configuration and the engaged ion is released into the cytoplasm. The cycle repeats at the previously disengaged aspartate in (4), (5), and (6) eventually resulting in a new power stroke and the return to stage (1).

By combining in silico characterization with in vivo testing we have identified coiled‐coil parameters and KIH features important for function. The initial alanine/isoleucine variant screen allowed the identification of BUDE profile features positively correlated with motility (Figures S1 and S2). The stabilization of the 28° conformation by residue M34 appeared to be an important factor for stator function and the height of the BUDE^max^ peak of each profile was also found to impact the motility of variants (Figure S2). By applying these classification criteria to a larger pool of in silico variants, we refined our definition of motility for *Ec*MotB variants by looking at the relative BUDE energies displayed by variants associated with a motility cluster (Figures 3B and S4) and the KIH arrangements shared by these variants (Figure S5). We found that motile variants displayed the 64°, 28°, and 56° configurations in ascending order of BUDE energy, suggesting that the coiled‐coil transitions between conformational states in this order in motile variants (Figure S4). We determined that the crucial KIH interaction at the 56° configuration that is mediated by residue F41 was missing in most of the non‐motile variants that were initially included in a “motile” cluster after parametrization (Figure S5). Coil‐coiled models of the WT MotB sequence indicate that M34 and F41 change side‐chain orientations between different configurations (Figure 3e,f). It is possible that the steric hindrance experienced by F41 during the register shift, or rotation of the coiled‐coil, is a major contributor to the height of the BUDE^max^ peak. The mechanisms described here might be shared by other extant bacterial stators (Figure 4). Taken together, these observations provide the rationale for our updated model of the stator gating cycle (Figure 5).

Among all the tested variants only some single mutants retained their ability to swim while all double‐ and triple‐mutant variants failed to swim, indicating a limited tolerance for mutagenesis for residues at the coil‐coil interface. Attempts at modulating the strength of CC interaction within the C‐terminal half of the 20‐residue coil resulted in non‐motile or poorly motile strains (M38A, F41A, and W45A) suggesting that “tightening” the coiled‐coil at the wrong knob could disrupt function entirely. KIH interactions appear to be important for stator function but predicting phenotypes from the modeled KIH interactions was difficult. For example, we tested a mutant with the intent to disrupt motility (M38G) by disrupting the KIH interactions between the coils in the 28° configuration (Figure S5) and confirmed the lack of motility in that variant (Figure S2B). Similarly, energy calculations indicated the M34I mutation could increase dimer stability for all analyzed coiled‐coil configurations, and this mutation yielded a motile variant. In contrast, mutating the central knob toward a new type of KIH interaction (M38I mutant) was predicted to stabilize only the 64° conformation. Yet this mutation abolished the KIH interaction at residue 38 in the 64° conformation and yielded a non‐motile variant.

A variant classification system helped to differentiate between motile and non‐motile stator classes and allowed to link BUDE energy calculations to KIH modeling. We defined two metrics, *f*1 and *f*2, to differentiate between the two classes and the effect of each of point mutant on motility. Once a variant was associated with a motile cluster, the KIH arrangement at the 28°, 56°, and 64° configurations was compared to WT to predict its phenotype. With this method we could correctly predict the motility of 28 out of 29 *Ec*MotB variants (Figure S5). The presence of KIH interactions at M34 (for the 28° configuration) and F41 (for 56° and 64°) were strong determinants of motility.

Using *f*1 as an indicator of functionality in a stator implies that the interaction is dominated by the knobs at position 34 and the conserved residue F41. We thus speculate that a transition between these two configurations is essential for the function of the stator. Previous work involving chemical crosslinking between MotB coiled‐coil residues suggested that the TM structure is flexible and able to exchange the residues at the CC interface during a rotation of ~36° (Braun & Blair, 2001).

The *f*1 and *f*2 parameters were useful also in describing and clustering extant MotB stalks. Indeed, it may be that these clusters represent families of MotB homologs that are related in their functional characteristics. Notably, *Vibrio alginolyticus* PomB and *E*. *coli* MotB, of which functional chimeras exist (Nishino et al., 2015), belong to the same cluster (green, Figure 4). These two stators are known to differ in their power source (Na^+^ and H^+^ respectively) and differ in the identity of their homologous residue 45 (L45 for Na^+^, W45 for H^+^) while showing similar 2D profiles. Another notable cluster is the one shared by *B*. *subtilis*, *C*. *sporogenes*, and *C*. *jejuni* (black, Figure 4). These three species are sources of the most detailed cryo‐EM structures of the MotA_5_B_2_ stator complex to date. It is possible that these complexes were more stable and thus easier to purify and resolve due to stronger MotB coil interaction than the *E*. *coli* or *V*. *alginolyticus* homologs.

We noted a trend linking the hydrophobicity of residue 38 with the BUDE^max^ magnitude. MotB clusters displaying F, L, or M at position 38 had a proportionally high BUDE^max^ peak in their profiles, with F38 being the highest and M38 the lowest (Figures 4B and 4C). The height of this peak could be related to the steric hindrance between the side chains of residue 38 and 41 of the two coils when rotating between two most stable configurations at 28° and 64°.

Elsewhere we have extensively examined large phylogenies of MotB (Islam et al., 2020). Here we calculated a small phylogeny which captured flagellar diversity to compare clusters derived from energetic profiles with clades obtained via phylogenetic analysis. In general, protein phylogenies should incorporate as much sequence information as possible rather than being truncated to a specific domain, in order that the calculation of maximum likelihood for common ancestry utilizes as much genetic information as possible (Engelhardt et al., 2011; Philippe et al., 2011). We assessed whether our energetic clustering, calculated only over the 20 TM residues of MotB and based on the *f*1 and *f*2 parameters, could reproduce phylogenetic clades that were calculated across the full‐length protein. Indeed, they did: our MotB classes from clustering similar energetic profiles closely matched clades from our phylogeny obtained using traditional tree‐building approaches (Figures 4A and S7). These results show that cluster‐based comparison of BUDE free energies can recapitulate phylogenetic clades and may indicate that there is evolutionary pressure driving towards the conservation of distinct stable coiled‐coil states. This clustering based on MotB‐MotB dimer stability could be further examined in greater detail, for example, with regard to species that have specific high‐torque operating environments (e.g., *Spirochaetes* sp.). These species typically have large rotors to generate larger torques (Beeby et al., 2016, 2020; Carroll & Liu, 2020; Guo & Liu, 2022), but there could be signatures conserved increased stator stability elsewhere that correlate with high‐torque or viscous operating conditions. Likewise, further studies in comparative phylogenetics could include MotA and MotB, as well as integrated holistic phylogenies across the rotor‐stator complex (Mo et al., 2022; San Martin et al., 2023). In conjunction with structural and modeling studies, these approaches could measure the variability of the evolutionary landscape and examine how molecular evolution led to adaptations at specific residues enabling the flagellar motor to adapt to new environments.

Based on the model proposed by Rieu et al. (2022), we describe a model of mechanochemical gating that includes the conformational changes in the MotB TM domain studied here (Figure 5). Our model also assumes that the rotation of the MotA pentamer can occur only if both catalytic aspartates in MotB are protonated. Pore‐lining residues from both subunits mediate the transport of the ion from the periplasm to the catalytic aspartate. We propose that a single protonated aspartate, engaging MotA with MotB, couples the synchronized rotation of both MotA_5_ and MotB_2_ by 36° clockwise, and the proton is then released into the cytoplasm after the entry of a second proton binding onto the other disengaged aspartate. Our model also assumes that the binding of a proton to the disengaged aspartate initiates the power stroke, which then occurs as both aspartates are protonated as postulated by Santiveri et al. (2020) and Rieu et al. (2022). The newly protonated site triggers a conformational change at the MotA‐MotB interface that promotes the engagement of the protonated aspartate with MotA at the second catalytic aspartate, the cytoplasmic pore exit site, as the complex rotates 36° clockwise relative to its TM axis.

In Mandadapu et al. (2015) model, a “kink” occurs in TM3 of MotA upon H^+^ binding to the aspartate and when MotB engages with TM3/4 of MotA this results in a swiveling motion of the TM3 of MotA that is the basis of the power stroke mechanism. Our data suggests that MotB might be coupled with the movement of MotA during its swiveling motion (a CW rotation) and switch between at least two of the stable configurations studied here, the 28° and 64° ones. Our model takes into account the 5:2 stoichiometry detected in structures and considers only one catalytic site at the time to be participating in a power stroke. The concerted CW rotation of the MotB TMs during the power stroke facilitates the alternating engagement of MotA subunits separated by 2/5th of a revolution, while maintaining MotB always in a parallel coiled‐coil state and without the need for vertical displacement of the MotB coils during the proton release stage. The activation of individual MotA subunits during the power stroke is reminiscent of the model proposed by Xing et al. but involving the alternating translocation of protons across the stator (Xing et al., 2006).

While we cannot determine the exact motions of the MotB TM dimer during the switch between its two most stable configurations, we suspect that the relative stability of the intermediate configurations (like the 56° one) is important for the completion of a 36° rotation of the interface angle of MotB. It is conceivable for the coiled‐coil dimer to tilt with respect to the MotA pentamer during this rotation to facilitate the engagement of the two subunits. It is also possible for other coiled‐coil parameters (pitch and radius), to change during this rotation which may stabilize the coiled‐coil at configurations considered inaccessible (like the 44° configuration). Mutations that further stabilize certain configurations, for example a more hydrophobic KIH at position 38 of the *Ec*MotB increasing the height of the BUDE^max^ peak (Figure 4), might interfere with the establishment of intermediate conformations which are necessarily crossed during the rotations between 28° and 64° configurations.

The function of the flagellar stator complex clearly depends not only on the pore‐lining residues between MotA and MotB and torque‐generating interactions between MotA and FliG (Hu, Popp, et al., 2022; Islam et al., 2022). Our overall conclusion is that it also depends on the degree of interaction between the two TM domains of MotB that assemble as a coiled‐coil at the core of the stator and the conformational states the dimer can access. The residues responsible for knob‐in‐hole interactions at the dimerization interface of *E*. *coli* MotB, and possibly other extant homologs, appear to tolerate only those mutations which do not shift their coiled‐coil energetics too far from wild‐type. Consideration of these energetics will improve our ability to engineer mutant MotB subunits, chimeric stators, or synthetic combinations of multiple flagellar motor proteins.

## 4 MATERIALS AND METHODS

### 4.1 Structural modeling of coiled‐coils

The *B*. *subtilis* MotA_5_B_2_ structure (PDB:6YSL) (Deme et al., 2020) was used as a reference to model the TM domain of *E*. *coli* MotB as coiled‐coil. We used ISAMBARD to create a specification whereby a parallel dimeric coiled‐coil backbone structure could be generated from three parameters: the interface angle, the radius, and the pitch. The coiled‐coil backbone structure for the amino acid sequence IAYADFMTAMMAFFLVMWLI (corresponding to the *E*. *coli* MotB residues 28–47) was optimized using the genetic algorithm evolutionary optimizer from ISAMBARD and SCWRL 4.0 for the optimization of side chain orientations to minimize the total energy of the system as calculated by the BUDE force field. Optimizations were performed using seven different initial interface angle values which corresponded to the seven possible registers of a coiled‐coil. The seven optimized coiled‐coil models were aligned to the resolved structure of the MotB dimer stalk in PDB structure 6YSL. The alignment revealed that the best initial interface angle that resulted in a good structural match after optimization to the MotB stalk was 51.4°, which corresponds to position *e* for the first residue in the amino acid sequence. The optimized parameter values were 4.54 Å radius, 232.03 Å pitch, and 74.85° interaction angle.

### 4.2 Calculation of the energy landscapes of the MotB coiled‐coils

We used in‐house Python scripts to generate a list of 221 sequence variants of the amino acid sequence IAY**A**DF**M**TAM**M**AF**F**LVM**W**LI where residues A31, M34, M38, F41, and W45 were systematically mutated to alanine in every permutation; separately the sequence was mutated with isoleucine (for residues A31, M38, and W45), asparagine (for residue M38), and leucine (for residues M34 and F41) in every permutation. These mutations follow coiled‐coil design principles to weaken or strengthen dimeric coiled‐coils. Finally, additional MotB mutants were added to the list (Table S1). For each sequence variant, we used ISAMBARD to generate coiled‐coil backbone structural models with radii values ranging from 4 to 6 Å (in 0.1 Å increments) and interface angle values ranging from 10 to 86° (in 4° increments). The pitch was kept constant to a value of to 150 Å. These ranges of values encompass the optimized parameters that best matched the reference structures. The sidechain orientations were optimized using SCWRL 4.0 as part of the ISAMBARD script. For each conformational variant, the total energy of the system was calculated using the BUDE forcefield. The 3D data sets (BUDE total energy as a function of interface angle and radius) were plotted as heatmaps using in‐house Python scripts and the Matplotlib library. The 2D datasets were generated by integrating the total energy values for a subset of the radii values (4–5 Å) which contained the stability wells and instability peaks of interest. The integrated total energy values were plotted as a function of the interface angles. In some analyses, we focused on four regions in the 3D dataset for comparison of the sequence variants (centered on combinations of radii and interface angles) and a representative energy value for each of the four regions was calculated by averaging two neighboring points from the 2D dataset (Figure 2c). For the identification of KIH interactions, MotB was modeled as a dimeric coiled‐coil starting at register *e* using CCBuilder 2.0, which evaluates the presence of KIHs using SOCKET (Walshaw & Woolfson, 2001).

### 4.3 *E. coli* strains, plasmids, and culture media

*E*. *coli* strain RP437 Δ*motAB* was prepared using the No‐Scar method (Reisch & Prather, 2015) using *E*. *coli* RP437 as parent strain. The scarless deletion of the *motA* and *motB* genes was confirmed by Sanger sequencing of colony‐PCR amplicons. MotA and MotB were expressed from plasmid pDB108 (Cm^+^) after induction with 0.1 mM Arabinose via the pBAD promoter. Liquid cell culturing was done using LB broth (85 mM NaCl, 0.5% yeast extract, and 1% Bacto tryptone). Point mutations in *motB* on the pDB108 plasmid were generated using the QuikChange™ technique and verified by Sanger sequencing. A list of primers used is provided in Table S2. Cells were cultured on agar plates composed of LB broth and 1% Bacto agar (BD Biosciences, USA). Swim plate cultures were performed on the same substrates adjusted for agar content (0.3% Bacto agar).

### 4.4 Single cell swimming assay

Free‐swimming cells were grown overnight in TB broth (85 mM NaCl, 1% Bacto tryptone) at 30°C to OD_600_ of ~0.5 then washed three times in 1 mL MB buffer (85 mM NaCl and 10 mM KPi, pH = 7.0) before resuspension in 500 μL of MB buffer and imaging in a tunnel slide. Time‐lapse videos were recorded at 20× magnification on a phase contrast microscope (Nikon). Time‐lapse videos were collected using a camera (Chameleon3 CM3, Point Grey Research) recording 20‐s‐long videos at 60 frames/s. A custom LabView software (Ishida et al., 2019) was used as previously reported to estimate specific rotational parameters of the tethered cells such as rotation frequency (speed), clockwise and counterclockwise bias, switching frequency, and speed of swimming cells.

## Supporting information


**Data S1.** Supporting information.
