# Supplementary material for: Tuning the stator subunit of the flagellar motor with coiled‐coil engineering
Source: Protein Sci. 2023 Dec 1;32(12):e4811. doi: 10.1002/pro.4811 (PMC10659934; doi:10.1002/pro.4811)
Supplement: Supplementary file 1 — Data S1. Supporting information. [file PRO-32-e4811-s001.pdf]

SUPPLEMENTARY MATERIAL

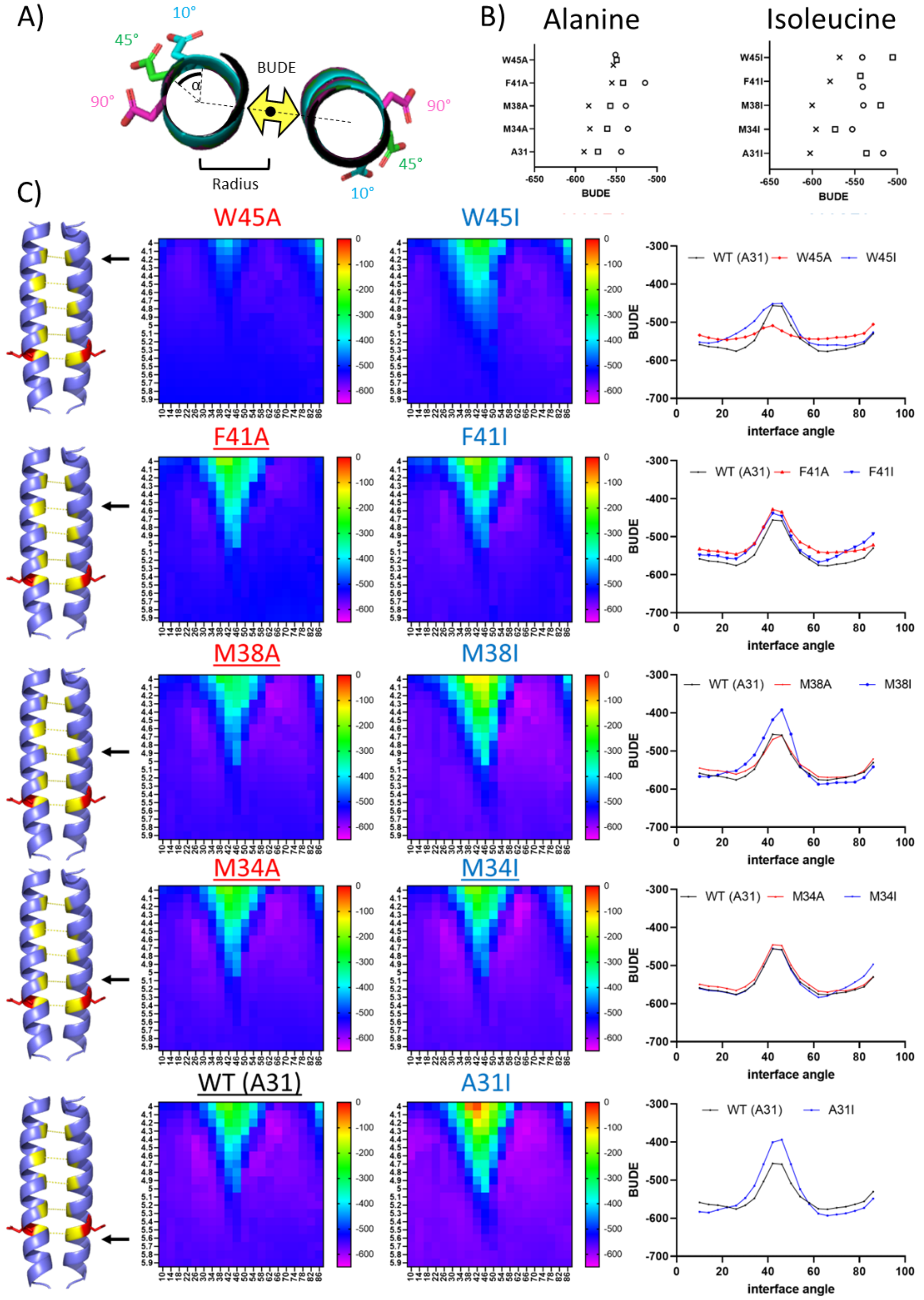

Supplementary Figure 1. BUDE modelling of MotB variants.

A) View from the top of the MotB dimeric coiled coil TM domain in different interface angles. The catalytic Aspartate D32 is highlighted on each monomer for interface angles 10°

(cyan), 45° (green) and 90° (pink). The coiled coil design parameters (Radius and Interface angle α) are schematically represented on the structure. The BUDE interaction energy is also represented as a two-headed yellow arrow in between the two alpha helices. B) Calculated BUDE values at the 28° (square), 56° (circle) and 64° (cross) configurations for Alanine (left) or Isoleucine (right) replacements at positions 31, 34, 38, 41, and 45 of WT *Ec*MotB. C) Schematic representation of the position of the amino acid residue being mutated to Ala or Ile along the TM domain of MotB (side view), heatmaps and 2D BUDE profiles of each single point mutant.

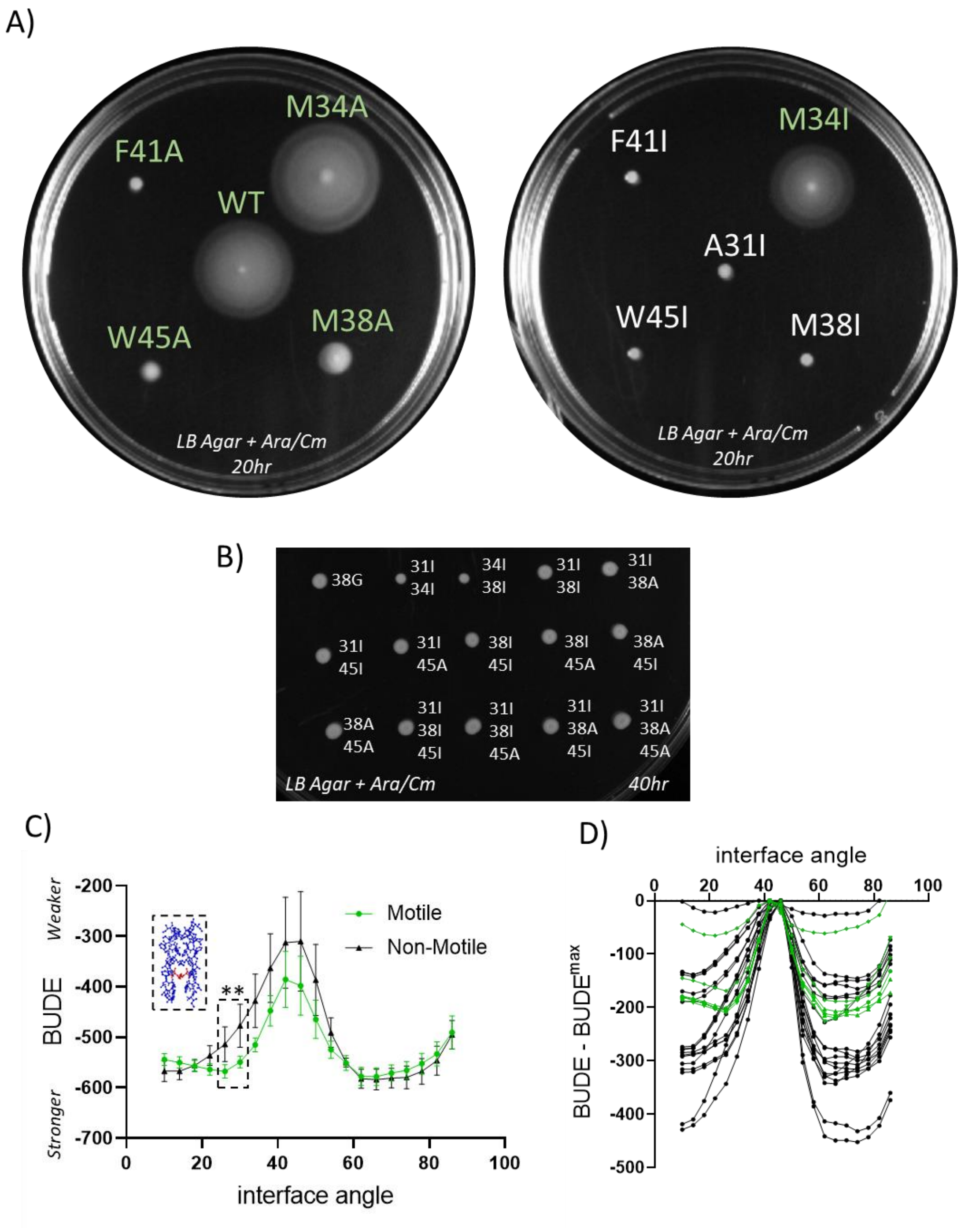

Supplementary Figure 2. Testing MotB variants in vivo in *E. coli.*

A-B) Swim plate swim assay of WT *Ec*MotB and all alanine (left) and isoleucine (right) coiled-coil variants initially screened. A pDB108 plasmid encoding each mutant variant along with WT MotA was expressed in *E. coli* Δ*mot*AB. The plate was incubated at 30°C for 20hr.

C) Average 2D BUDE plots of motile (green) vs non-motile (black) MotB variants. The KIH present in MotB in the 28° configuration is highlighted in red on the atomic model in the

dashed box. Bars indicate Mean + SD. D) Individual 2D BUDE plots of motile (green) vs non-motile (black) MotB variants, translated according to their respective $BUDE^{max}$ values.

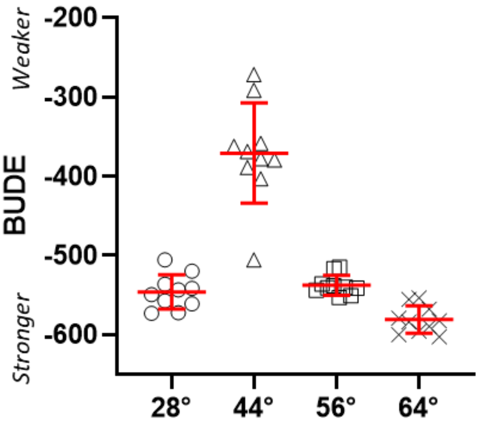

	28°	56°	64°	44°
Number of values	10	10	10	10
Minimum	-573.1	-553.0	-602.4	-505.4
Maximum	-505.5	-514.7	-553.6	-271.5
Range	67.60	38.30	48.80	233.9
Mean	-545.9	-537.5	-580.9	-370.7
Std. Deviation	21.73	12.71	17.32	63.26
Std. Error of Mean	6.872	4.020	5.477	20.00

Supplementary Figure 3. Variability of BUDE values at each configuration hotspot.

Average BUDE values at each hotspot configuration for WT and all single point mutants (left). Error bars indicate Mean and Standard Deviation. Complete descriptive statistics for the plotted points are provided on the right. The standard deviation of the 56° configuration is highlighted in red.

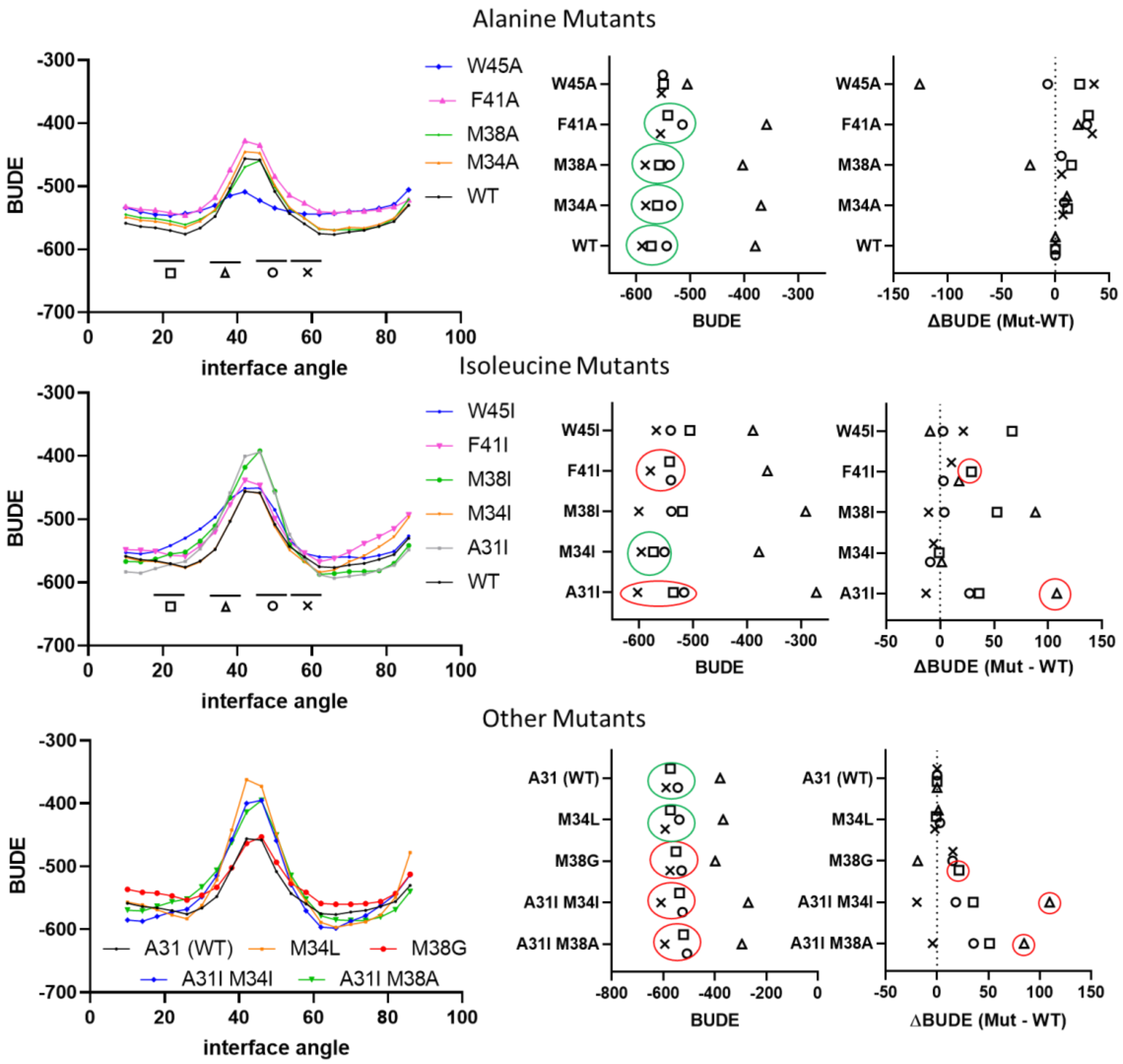

Supplementary Figure 4. BUDE profile features correlated with motile and non-motile phenotypes.

Two-dimensional BUDE profiles (left), BUDE values (centre) and ΔBUDE (right) compared to WT, calculated at the 28° (square), 44° (triangle), 56° (circle) and 64° (cross) configurations for Alanine (top) or Isoleucine (middle) and other (bottom) replacements at positions 31, 34, 38, 41, and 45 of WT EcMotB. BUDE values graphs (centre): Green circles indicate motile variants displaying a cross-square-circle pattern of BUDE values, red circles indicate non-motile variants also displaying the same pattern. ΔBUDE graphs (right): Red circles indicate destabilized conformations (28° and 44°) in non-motile variants displaying the cross-square-circle pattern that may be causing the loss-of motility

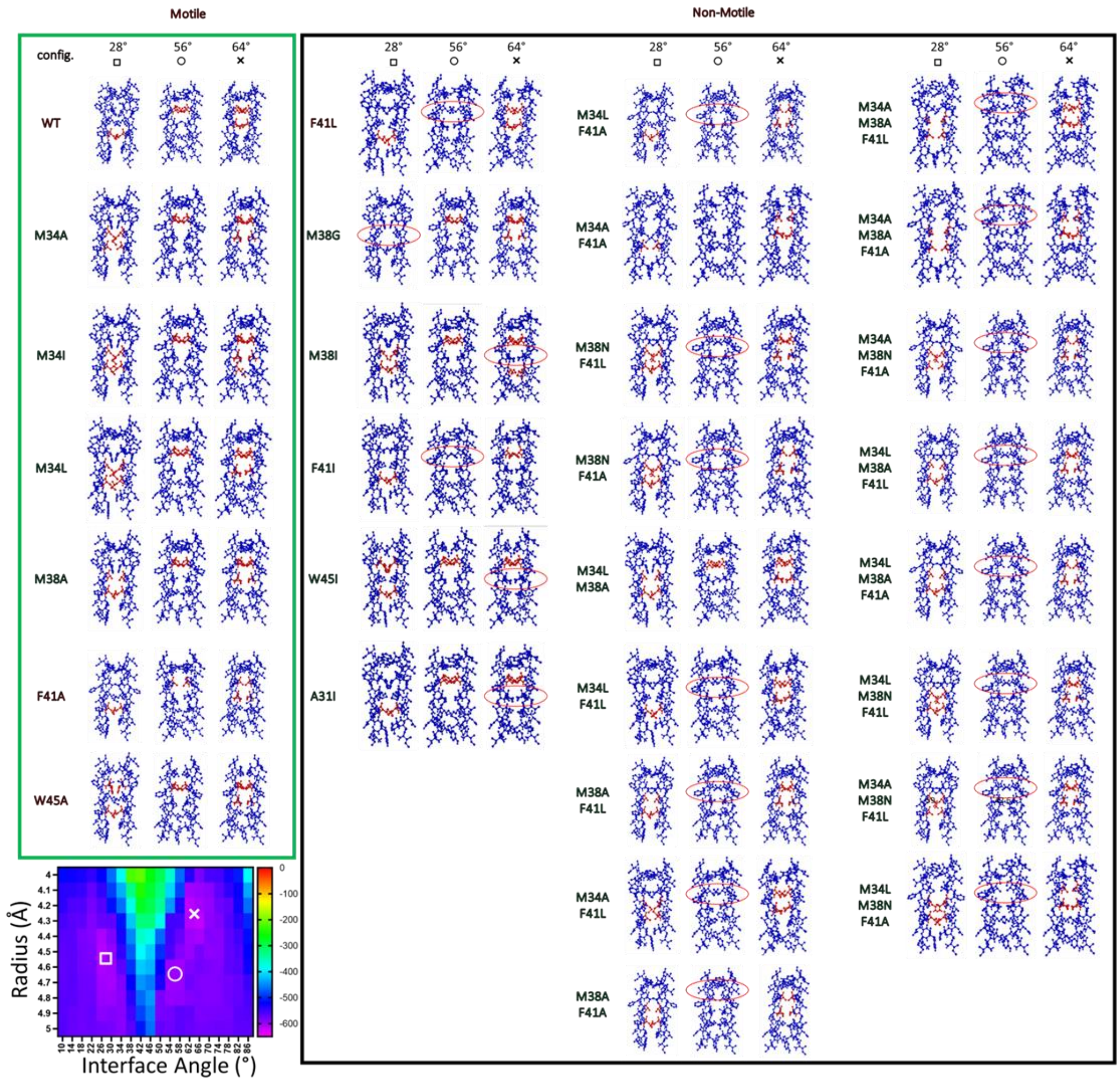

Supplementary Figure 5. CCBuilder models of all coiled-coil variants tested in *E. coli*.

A CCBuilder model of each variant in each of the three radius/interface angle configurations marked by square (28°) circle (56°) and cross (64°) on the BUDE plot shown on the bottom-left. All motile variants are included in the green box while all non-motile variants are included in the black box. Red ellipses mark the missing KIH interactions in the non-motile variants.

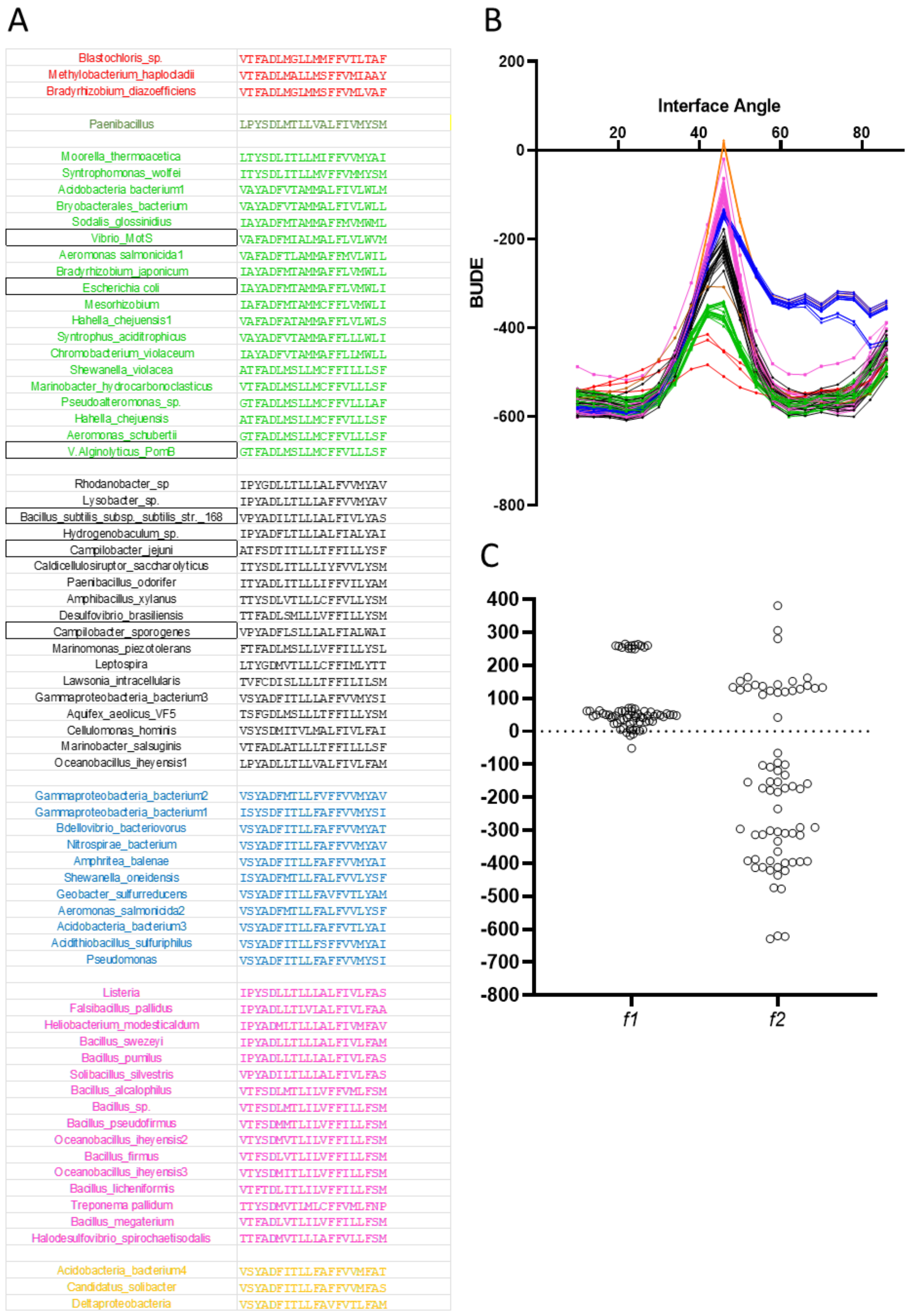

Supplementary Figure 6. BUDE profile analysis of extant MotB homologs.

A) List of species and respective 20 amino acid homologous sequence included in the analysis of extant MotB's, color-coded according to the clusters presented in B and Fig. 3A. Notable species in the list are also highlighted by a black box. B) 2D Bude plots for all species described in A. C) Parameters *f1* and *f2* plotted side by side for all profiles describe in B.

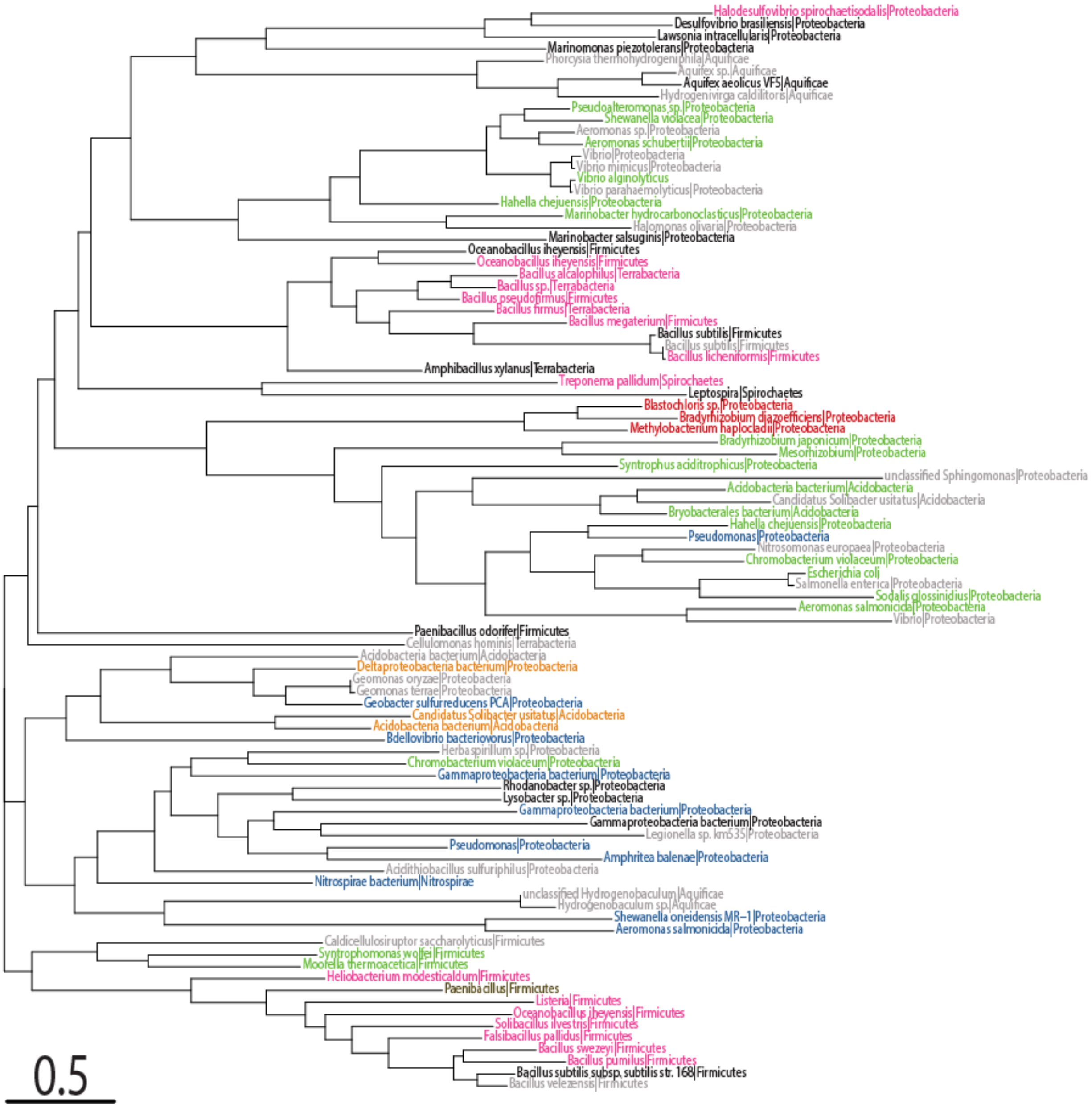

Supplementary Figure 7. Phylogeny of MotB of 91 bacterial species.

Tips are coloured according to the clustering shown in Fig. 4 and SI Fig. 6. Grey labels indicate species that were not included in the analysis.

Supplementary Table 1 – List of MotB mutants for *in silico* modelling. Variants tested *in vivo* are bolded.

MotB Variant	20 residue amino acid sequence
**WT *Ec*MotB**	IAYADFMTAMMAFFLVMWLI
**M34I**	IAYADFITAMMAFFLVMWLI
**M38G**	IAYADFMTAMGAFFLVMWLI
**F41I**	IAYADFMTAMMAFILVMWLI
**A31I M34I**	IAYIDFITAMMAFFLVMWLI
**M34I M38I**	IAYADFITAMIAFFLVMWLI
**W45I**	IAYADFMTAMMAFFLVMILI
**W45A**	IAYADFMTAMMAFFLVMALI
**F41L**	IAYADFMTAMMAFLLVMWLI
F41L W45I	IAYADFMTAMMAFLLVMILI
F41L W45A	IAYADFMTAMMAFLLVMALI
**F41A**	IAYADFMTAMMAFALVMWLI
F41A W45I	IAYADFMTAMMAFALVMILI
F41A W45A	IAYADFMTAMMAFALVMALI
**M38I**	IAYADFMTAMIAFFLVMWLI
**M38I W45I**	IAYADFMTAMIAFFLVMILI
**M38I W45A**	IAYADFMTAMIAFFLVMALI
M38I F41L	IAYADFMTAMIAFLLVMWLI
M38I F41L W45I	IAYADFMTAMIAFLLVMILI
M38I F41L W45A	IAYADFMTAMIAFLLVMALI
M38I F41A	IAYADFMTAMIAFALVMWLI
M38I F41A W45I	IAYADFMTAMIAFALVMILI
M38I F41A W45A	IAYADFMTAMIAFALVMALI
M38N	IAYADFMTAMNAFFLVMWLI
M38N W45I	IAYADFMTAMNAFFLVMILI
M38N W45A	IAYADFMTAMNAFFLVMALI
M38N F41L	IAYADFMTAMNAFLLVMWLI
M38N F41L W45I	IAYADFMTAMNAFLLVMILI
M38N F41L W45A	IAYADFMTAMNAFLLVMALI
M38N F41A	IAYADFMTAMNAFALVMWLI
M38N F41A W45I	IAYADFMTAMNAFALVMILI
M38N F41A W45A	IAYADFMTAMNAFALVMALI
**M38A**	IAYADFMTAMAAFFLVMWLI
**M38A W45I**	IAYADFMTAMAAFFLVMILI
**M38A W45A**	IAYADFMTAMAAFFLVMALI
M38A F41L	IAYADFMTAMAAFLLVMWLI
M38A F41L W45I	IAYADFMTAMAAFLLVMILI
M38A F41L W45A	IAYADFMTAMAAFLLVMALI
M38A F41A	IAYADFMTAMAAFALVMWLI
M38A F41A W45I	IAYADFMTAMAAFALVMILI
M38A F41A W45A	IAYADFMTAMAAFALVMALI
**M34L**	IAYADFLTAMMAFFLVMWLI

M34L W45I	IAYADFLTAMMAFFLVMILI
M34L W45A	IAYADFLTAMMAFFLVMALI
**M34L F41L**	IAYADFLTAMMAFLLVMWLI
M34L F41L W45I	IAYADFLTAMMAFLLVMILI
M34L F41L W45A	IAYADFLTAMMAFLLVMALI
M34L F41A	IAYADFLTAMMAFALVMWLI
M34L F41A W45I	IAYADFLTAMMAFALVMILI
M34L F41A W45A	IAYADFLTAMMAFALVMALI
M34L M38I	IAYADFLTAMIAFFLVMWLI
M34L M38I W45I	IAYADFLTAMIAFFLVMILI
M34L M38I W45A	IAYADFLTAMIAFFLVMALI
M34L M38I F41L	IAYADFLTAMIAFLLVMWLI
M34L M38I F41L W45I	IAYADFLTAMIAFLLVMILI
M34L M38I F41L W45A	IAYADFLTAMIAFLLVMALI
M34L M38I F41A	IAYADFLTAMIAFALVMWLI
M34L M38I F41A W45I	IAYADFLTAMIAFALVMILI
M34L M38I F41A W45A	IAYADFLTAMIAFALVMALI
M34L M38N	IAYADFLTAMNAFFLVMWLI
M34L M38N W45I	IAYADFLTAMNAFFLVMILI
M34L M38N W45A	IAYADFLTAMNAFFLVMALI
M34L M38N F41L	IAYADFLTAMNAFLLVMWLI
M34L M38N F41L W45I	IAYADFLTAMNAFLLVMILI
M34L M38N F41L W45A	IAYADFLTAMNAFLLVMALI
**M34L M38N F41A**	IAYADFLTAMNAFALVMWLI
M34L M38N F41A W45I	IAYADFLTAMNAFALVMILI
M34L M38N F41A W45A	IAYADFLTAMNAFALVMALI
**M34L M38A**	IAYADFLTAMAAFFLVMWLI
M34L M38A W45I	IAYADFLTAMAAFFLVMILI
M34L M38A W45A	IAYADFLTAMAAFFLVMALI
M34L M38A F41L	IAYADFLTAMAAFLLVMWLI
M34L M38A F41L W45I	IAYADFLTAMAAFLLVMILI
M34L M38A F41L W45A	IAYADFLTAMAAFLLVMALI
M34L M38A F41A	IAYADFLTAMAAFALVMWLI
M34L M38A F41A W45I	IAYADFLTAMAAFALVMILI
M34L M38A F41A W45A	IAYADFLTAMAAFALVMALI
**M34A**	IAYADFATAMMAFFLVMWLI
M34A W45I	IAYADFATAMMAFFLVMILI
M34A W45A	IAYADFATAMMAFFLVMALI
**M34A F41L**	IAYADFATAMMAFLLVMWLI
M34A F41L W45I	IAYADFATAMMAFLLVMILI
M34A F41L W45A	IAYADFATAMMAFLLVMALI
M34A F41A	IAYADFATAMMAFALVMWLI
M34A F41A W45I	IAYADFATAMMAFALVMILI
M34A F41A W45A	IAYADFATAMMAFALVMALI
M34A M38I	IAYADFATAMIAFFLVMWLI
M34A M38I W45I	IAYADFATAMIAFFLVMILI

M34A M38I W45A	IAYADFATAMIAFFLVMALI
M34A M38I F41L	IAYADFATAMIAFLLVMWLI
M34A M38I F41L W45I	IAYADFATAMIAFLLVMILI
M34A M38I F41L W45A	IAYADFATAMIAFLLVMALI
M34A M38I F41A	IAYADFATAMIAFALVMWLI
M34A M38I F41A W45I	IAYADFATAMIAFALVMILI
M34A M38I F41A W45A	IAYADFATAMIAFALVMALI
M34A M38N	IAYADFATAMNAFFLVMWLI
M34A M38N W45I	IAYADFATAMNAFFLVMILI
M34A M38N W45A	IAYADFATAMNAFFLVMALI
M34A M38N F41L	IAYADFATAMNAFLLVMWLI
M34A M38N F41L W45I	IAYADFATAMNAFLLVMILI
M34A M38N F41L W45A	IAYADFATAMNAFLLVMALI
M34A M38N F41A	IAYADFATAMNAFALVMWLI
M34A M38N F41A W45I	IAYADFATAMNAFALVMILI
M34A M38N F41A W45A	IAYADFATAMNAFALVMALI
M34A M38A	IAYADFATAMAAFFLVMWLI
M34A M38A W45I	IAYADFATAMAAFFLVMILI
M34A M38A W45A	IAYADFATAMAAFFLVMALI
M34A M38A F41L	IAYADFATAMAAFLLVMWLI
M34A M38A F41L W45I	IAYADFATAMAAFLLVMILI
M34A M38A F41L W45A	IAYADFATAMAAFLLVMALI
M34A M38A F41A	IAYADFATAMAAFALVMWLI
M34A M38A F41A W45I	IAYADFATAMAAFALVMILI
M34A M38A F41A W45A	IAYADFATAMAAFALVMALI
**A31I**	IAYIDFMTAMMAFFLVMWLI
**A31I W45I**	IAYIDFMTAMMAFFLVMILI
**A31I W45A**	IAYIDFMTAMMAFFLVMALI
A31I F41L	IAYIDFMTAMMAFLLVMWLI
A31I F41L W45I	IAYIDFMTAMMAFLLVMILI
A31I F41L W45A	IAYIDFMTAMMAFLLVMALI
A31I F41A	IAYIDFMTAMMAFALVMWLI
A31I F41A W45I	IAYIDFMTAMMAFALVMILI
A31I F41A W45A	IAYIDFMTAMMAFALVMALI
**A31I M38I**	IAYIDFMTAMIAFFLVMWLI
**A31I M38I W45I**	IAYIDFMTAMIAFFLVMILI
**A31I M38I W45A**	IAYIDFMTAMIAFFLVMALI
A31I M38I F41L	IAYIDFMTAMIAFLLVMWLI
A31I M38I F41L W45I	IAYIDFMTAMIAFLLVMILI
A31I M38I F41L W45A	IAYIDFMTAMIAFLLVMALI
A31I M38I F41A	IAYIDFMTAMIAFALVMWLI
A31I M38I F41A W45I	IAYIDFMTAMIAFALVMILI
A31I M38I F41A W45A	IAYIDFMTAMIAFALVMALI
A31I M38N	IAYIDFMTAMNAFFLVMWLI
A31I M38N W45I	IAYIDFMTAMNAFFLVMILI
A31I M38N W45A	IAYIDFMTAMNAFFLVMALI

A31I M38N F41L	IAYIDFMTAMNAFLLVMWLI
A31I M38N F41L W45I	IAYIDFMTAMNAFLLVMILI
A31I M38N F41L W45A	IAYIDFMTAMNAFLLVMALI
A31I M38N F41A	IAYIDFMTAMNAFALVMWLI
A31I M38N F41A W45I	IAYIDFMTAMNAFALVMILI
A31I M38N F41A W45A	IAYIDFMTAMNAFALVMALI
**A31I M38A**	IAYIDFMTAMAAFFLVMWLI
**A31I M38A W45I**	IAYIDFMTAMAAFFLVMILI
**A31I M38A W45A**	IAYIDFMTAMAAFFLVMALI
A31I M38A F41L	IAYIDFMTAMAAFLLVMWLI
A31I M38A F41L W45I	IAYIDFMTAMAAFLLVMILI
A31I M38A F41L W45A	IAYIDFMTAMAAFLLVMALI
A31I M38A F41A	IAYIDFMTAMAAFALVMWLI
A31I M38A F41A W45I	IAYIDFMTAMAAFALVMILI
A31I M38A F41A W45A	IAYIDFMTAMAAFALVMALI
A31I M34L	IAYIDFLTAMMAFFLVMWLI
A31I M34L W45I	IAYIDFLTAMMAFFLVMILI
A31I M34L W45A	IAYIDFLTAMMAFFLVMALI
A31I M34L F41L	IAYIDFLTAMMAFLLVMWLI
A31I M34L F41L W45I	IAYIDFLTAMMAFLLVMILI
A31I M34L F41L W45A	IAYIDFLTAMMAFLLVMALI
A31I M34L F41A	IAYIDFLTAMMAFALVMWLI
A31I M34L F41A W45I	IAYIDFLTAMMAFALVMILI
A31I M34L F41A W45A	IAYIDFLTAMMAFALVMALI
A31I M34L M38I	IAYIDFLTAMIAFFLVMWLI
A31I M34L M38I W45I	IAYIDFLTAMIAFFLVMILI
A31I M34L M38I W45A	IAYIDFLTAMIAFFLVMALI
A31I M34L M38I F41L	IAYIDFLTAMIAFLLVMWLI
A31I M34L M38I F41L W45I	IAYIDFLTAMIAFLLVMILI
A31I M34L M38I F41L W45A	IAYIDFLTAMIAFLLVMALI
A31I M34L M38I F41A	IAYIDFLTAMIAFALVMWLI
A31I M34L M38I F41A W45I	IAYIDFLTAMIAFALVMILI
A31I M34L M38I F41A W45A	IAYIDFLTAMIAFALVMALI
A31I M34L M38N	IAYIDFLTAMNAFFLVMWLI
A31I M34L M38N W45I	IAYIDFLTAMNAFFLVMILI
A31I M34L M38N W45A	IAYIDFLTAMNAFFLVMALI
A31I M34L M38N F41L	IAYIDFLTAMNAFLLVMWLI
A31I M34L M38N F41L W45I	IAYIDFLTAMNAFLLVMILI
A31I M34L M38N F41L W45A	IAYIDFLTAMNAFLLVMALI
A31I M34L M38N F41A	IAYIDFLTAMNAFALVMWLI
A31I M34L M38N F41A W45I	IAYIDFLTAMNAFALVMILI
A31I M34L M38N F41A W45A	IAYIDFLTAMNAFALVMALI
A31I M34L M38A	IAYIDFLTAMAAFFLVMWLI
A31I M34L M38A W45I	IAYIDFLTAMAAFFLVMILI
A31I M34L M38A W45A	IAYIDFLTAMAAFFLVMALI
A31I M34L M38A F41L	IAYIDFLTAMAAFLLVMWLI

A31I M34L M38A F41L W45I	IAYIDFLTAMAAFLLVMILI
A31I M34L M38A F41L W45A	IAYIDFLTAMAAFLLVMALI
A31I M34L M38A F41A	IAYIDFLTAMAAFALVMWLI
A31I M34L M38A F41A W45I	IAYIDFLTAMAAFALVMILI
A31I M34L M38A F41A W45A	IAYIDFLTAMAAFALVMALI
A31I M34A	IAYIDFATAMMAFFLVMWLI
A31I M34A W45I	IAYIDFATAMMAFFLVMILI
A31I M34A W45A	IAYIDFATAMMAFFLVMALI
A31I M34A F41L	IAYIDFATAMMAFLLVMWLI
A31I M34A F41L W45I	IAYIDFATAMMAFLLVMILI
A31I M34A F41L W45A	IAYIDFATAMMAFLLVMALI
A31I M34A F41A	IAYIDFATAMMAFALVMWLI
A31I M34A F41A W45I	IAYIDFATAMMAFALVMILI
A31I M34A F41A W45A	IAYIDFATAMMAFALVMALI
A31I M34A M38I	IAYIDFATAMIAFFLVMWLI
A31I M34A M38I W45I	IAYIDFATAMIAFFLVMILI
A31I M34A M38I W45A	IAYIDFATAMIAFFLVMALI
A31I M34A M38I F41L	IAYIDFATAMIAFLLVMWLI
A31I M34A M38I F41L W45I	IAYIDFATAMIAFLLVMILI
A31I M34A M38I F41L W45A	IAYIDFATAMIAFLLVMALI
A31I M34A M38I F41A	IAYIDFATAMIAFALVMWLI
A31I M34A M38I F41A W45I	IAYIDFATAMIAFALVMILI
A31I M34A M38I F41A W45A	IAYIDFATAMIAFALVMALI
A31I M34A M38N	IAYIDFATAMNAFFLVMWLI
A31I M34A M38N W45I	IAYIDFATAMNAFFLVMILI
A31I M34A M38N W45A	IAYIDFATAMNAFFLVMALI
A31I M34A M38N F41L	IAYIDFATAMNAFLLVMWLI
A31I M34A M38N F41L W45I	IAYIDFATAMNAFLLVMILI
A31I M34A M38N F41L W45A	IAYIDFATAMNAFLLVMALI
A31I M34A M38N F41A	IAYIDFATAMNAFALVMWLI
A31I M34A M38N F41A W45I	IAYIDFATAMNAFALVMILI
A31I M34A M38N F41A W45A	IAYIDFATAMNAFALVMALI
A31I M34A M38A	IAYIDFATAMAAFFLVMWLI
A31I M34A M38A W45I	IAYIDFATAMAAFFLVMILI
A31I M34A M38A W45A	IAYIDFATAMAAFFLVMALI
A31I M34A M38A F41L	IAYIDFATAMAAFLLVMWLI
A31I M34A M38A F41L W45I	IAYIDFATAMAAFLLVMILI
A31I M34A M38A F41L W45A	IAYIDFATAMAAFLLVMALI
A31I M34A M38A F41A	IAYIDFATAMAAFALVMWLI
A31I M34A M38A F41A W45I	IAYIDFATAMAAFALVMILI
A31I M34A M38A F41A W45A	IAYIDFATAMAAFALVMALI

Supplementary Table 2 – List of primers for MotB mutagenesis

Primer name	Sequence (5' to 3')

A31I -Fw	gcacatggatcgtggaagattgcttatatcgactttatgactgcg
A31I-Rv	cgcagtcataaagtcgatataagcaatcttccacgatccatgtgc
M34A-Fw	agattgcttatgccgactttgcgactgcgatgatggccttt
M34A-Rv	aaaggccatcatcgcagtcgcaaagtcggcataagcaatct
M34I-Fw	ggaagattgcttatgccgactttataactgcgatgatgg
M34I-Rv	ccatcatcgcagttataaagtcggcataagcaatcttcc
M34L-Fw	ggaagattgcttatgccgactttttgactgcgatga
M34L-Rv	tcatcgcagtcaaaaagtcggcataagcaatcttcc
M38I-Fw	ccgactttatgactgcgatgatagcctttttttctgg
M38I-Rv	ccagaaaaaaaggctatcatcgcagtcataaagtcgg
M38A-Fw	cgactttatgactgcgatggcggcctttttttctggtgatg
M38A-Rv	catcaccagaaaaaaaggccgccatcgcagtcataaagtcg
M38G-Fw	ccgactttatgactgcgatgatagcctttttttctgg
M38G-Rv	ccagaaaaaaaggctatcatcgcagtcataaagtcgg
F41A-Fw	ttatgactgcgatgatggcctttgctctggtgatgtggct
F41A-Rv	agccacatcaccagagcaaaggccatcatcgcagtcataa
F41I-Fw	ctgcgatgatggcctttattctggtgatgtggct
F41I-Rv	agccacatcaccagaataaaggccatcatcgcag
F41L-Fw	ctgcgatgatggcctttttactggtgatgtggc
F41L-Rv	gccacatcaccagtaaaaaggccatcatcgcag
W45A-Fw	gcctttttttctggtgatggcgctgatctccatctccag
W45A-Rv	ctggagatggagatcagcgccatcaccagaaaaaaaggc
W45I-Fw	tggcctttttttctggtgatgatactgatctccatctccagccc
W45I-Rv	gggctggagatggagatcagtatcatcaccagaaaaaaaggcca
A31I/M34I-Fw	gtggaagattgcttatatcgactttataactgcgatgatggc
A31I/M34I-Rv	gccatcatcgcagttataaagtcgatataagcaatcttccac
M34I/M38I-Fw	ggaagattgcttatgccgactttataactgcgatgatag
M34I/M38I-Rv	ctatcatcgcagttataaagtcggcataagcaatcttcc
